# An Entropy-Based Failure Prediction Model for the Creep and Fatigue of Metallic Materials

**DOI:** 10.3390/e21111104

**Published:** 2019-11-12

**Authors:** Jundong Wang, Yao Yao

**Affiliations:** 1 School of Mechanics, Civil Engineering and Architecture, Northwestern Polytechnical University, Xi’an 710072, China; 2 School of Civil Engineering, Xi’an University of Architecture and Technology, Xi’an 710055, China

**Keywords:** entropy increase rate, creep strain, damage mechanics, fatigue, metallic material

## Abstract

It is well accepted that the second law of thermodynamics describes an irreversible process, which can be reflected by the entropy increase. Irreversible creep and fatigue damage can also be represented by a gradually increasing damage parameter. In the current study, an entropy-based failure prediction model for creep and fatigue is proposed based on the Boltzmann probabilistic entropy theory and continuum damage mechanics. A new method to determine the entropy increment rate for creep and fatigue processes is proposed. The relationship between entropy increase rate during creep process and normalized creep failure time is developed and compared with the experimental results. An empirical formula is proposed to describe the evolution law of entropy increase rate and normalized creep time. An entropy-based model is developed to predict the change of creep strain during the damage process. Experimental results of metals and alloys with different stresses and at different temperatures are adopted to verify the proposed model. It shows that the theoretical predictions agree well with experimental data.

## 1. Introduction

In the past decades, fatigue of materials has been investigated extensively with respect to crack nucleation, propagation, and life prediction under cyclic loading. Numerous theoretical models have been proposed based on statistics or empirical methods. The models adopted in industry are usually empirical and the physical mechanism of fatigue damage and life prediction still requires further study.

Creep is time-dependent and can be accelerated by increasing of the stress and temperature. It is one of the common damage modes in engineering such as turbine blades, thermal plants, and thermonuclear installations, especially at high-temperature conditions. The creep deformation can emerge even when the applied stress is below the elastic limit, which is more pronounced when the ambient temperature approaching the melting point of materials. Generally, the creep deformation behavior is distinguished by three stages: The creep strain rate decreases constantly in the first stage; the creep strain rate keeps almost constant in the second stage; and in the third stage, the creep rate increases rapidly until failure. The potential physical mechanism of different stages can be explained by the dislocation theory for metallic materials [1,2,3,4,5,6]. The dislocation density changes during the creep process, micro-voids nucleate in the first stage, and the coalescence and propagation mechanism of micro-voids occur in the second and the third stages simultaneously, which leads to the final fracture.

The relationship between creep strain rate and creep life was widely investigated theoretically and experimentally for different engineering materials. Monkman and Grant [7] proposed a model to describe the evolution law of steady creep strain rate and creep life and it was successfully applied to metallic materials. This model was subsequently modified by considering the damage parameters to describe the creep behavior [8,9,10,11]. Dyson and Gibbons [12] related the normalized creep strain and time by considering the applied stress and damage variable during the creep process. The proposed model linked the strain with time by introducing a damage variable. One of the widely adopted creep life prediction models for carbon steel is proposed by Fields [13]. A power law is applied to relate the creep stress and time. The parameters in this model can be determined from experiments [14].

The applications of thermodynamic methodology to contact problems [15,16,17] introduced entropy into solid mechanics. The specific entropy was applied for the complex systems under mechanical fatigue, thermal loading, friction, and wear conditions [18,19,20]. The entropy increase rate was studied under the framework of Boltzmann probabilistic theory and continuum damage mechanics [21]. A low-cycle fatigue life prediction model was proposed with respect to the entropy increase rate [21]. Based on the second law of thermodynamics, creep damage process is also irreversible, which can be represented by the increasing of entropy in the entire creep life.

In the current study, the entropy increase rate model and its application in fatigue life prediction is reviewed. Then, the entropy increase rate model is applied to describe the creep behavior. The relationship between entropy increase rate and normalized creep time is investigated based on the experimental analysis. A unified entropy increase trend was observed for metallic materials under different experimental conditions. An empirical formula is then proposed to describe the entropy increase rate during creep process. An entropy-based creep life prediction model is obtained by solving an ordinary differential equation. Comparison with experimental data indicates that the proposed model can accurately predict creep behavior of metallic materials with different stresses and temperatures. 

## 2. The Change Regulation of Entropy Increase Rate during Degeneration Process

For ideal gas system, Boltzmann [22] defined a precise relationship between the disorder state and entropy:

(1) $$S=k_{0}\ln\left(W\right)$$

where $k_{0}$ is the Boltzmann constant and *W* is the disorder state parameter, which represents the probability of the system to exist in the current state relative to all the possible states. Although it is difficult to determine the value of *W*, Equation (1) provides an approach to determine the disorder of molecular thermal motion in the system. 

The relation between entropy per unit mass and the disorder parameter was improved by Basaran et al. [20]. The disorder state parameter *W* was defined as a function of the entropy *S*, Avogadro constant, and the specific mass $m_{s}$: $W=\exp\left(Sm_{s}/N_{0}\right)$. Basaran et al. [23,24,25,26,27] proposed a relation between the entropy per unit mass and disorder state parameter; a damage law is then developed, which links the damage parameter D and entropy S:

(2) $$D=D_{cr}\frac{W-W_{0}}{W}=D_{cr}\left[1-\exp\left(-\frac{m_{s}\left(S-S_{0}\right)}{N_{0}k_{0}}\right)\right]$$

where $W_{0}$ represents the disorder corresponding to the initial state of the continuous medium with entropy $S_{0}$ and $D_{cr}$ is the critical value of damage approaching final failure.

The degeneration process, such as creep and fatigue, is not only a damage process, but also irreversible, which is consistent with the second law of thermodynamics. Although the damage variable is an artificially defined quantity in the viewpoint of solid mechanics, it has the same trend for entropy without decreasing. Determination of damage parameters requires different physical quantities including the elasticity modulus, micro-hardness, density, and electrical resistance etc. Variation of these physical quantities represents the corresponding microstructure change of material. The variation of entropy during the damage process represents the logarithm change of the molecular configurations [22]. Both quantities represent the microstructure change in different states, one for an outward manifestation and the other for the essential molecular configurations. In addition, the entropy and damage parameters are all monotonically changed during the degeneration process. Hence, it is possible to establish a connection between the damage variable and entropy.

A classical damage rate model was proposed by Bonora [28] based on the continuous damage mechanics and the plastic part of the Ranberg–Osgood power law:

(3) $$\dot{D}=-\dot{\lambda }\frac{\partial \Phi }{\partial Y}=\frac{K^{2}}{2Ea_{0}}\frac{{\left(D_{cr}-D\right)}^{\frac{\alpha -1}{\alpha }}}{p}f\left(\frac{\sigma_{m}}{\sigma_{eq}}\right)\frac{\dot{\lambda }}{1-D}$$

where *K* and $a_{0}$ are the material constants, $\dot{\lambda }$ is plastic multiplier, α is the damage exponent and can be obtained by determining the change of elastic modulus during the damage process, $f\left(\frac{\sigma_{m}}{\sigma_{eq}}\right)$ is a factor, and for uniaxial loading $f\left(\frac{\sigma_{m}}{\sigma_{eq}}\right)=1$. By applying the relation between plastic multiplier $\dot{\lambda }$ and cumulative plastic strain rate $\dot{p}$: $\dot{\lambda }=\dot{p}\cdot \left(1-D\right)$, Equation (3) can be written in another form:

(4) $$\dot{D}=\frac{K^{2}}{2Ea_{0}}\frac{{\left(D_{cr}-D\right)}^{\frac{\alpha -1}{\alpha }}}{p}f\left(\frac{\sigma_{m}}{\sigma_{eq}}\right)\frac{\dot{p}}{p}$$

The damage variable D has two threshold values, $D_{0}$ and $D_{cr}$. The threshold $D_{0}$ represents the initial value of damage variable presented in material microstructure or the value at the beginning of creep or fatigue damage accumulation. The threshold $D_{cr}$ is the critical value of damage variable when creep or fatigue failure occurs. The corresponding cumulative plastic strain for $D=D_{0}$ and $D=D_{cr}$ are $p_{th}$ and $p_{cr}$, respectively. Integrating Equation (4) between $\left[D,D_{cr}\right]$ and $\left[p,p_{cr}\right]$ gives:

(5) $${\left(D-D_{cr}\right)}^{1/\alpha }=\frac{K^{2}}{2Ea_{0}\alpha }ln\left(\frac{p_{cr}}{p}\right)f\left(\frac{\sigma_{m}}{\sigma_{eq}}\right).$$

Based on the plasticity damage dissipation potentials in Equation (2), an entropy increasing rate model for uniaxial state was proposed (detailed derivation can be found in [21]):

(6) $$\dot{S}=\frac{N_{0}k_{0}\alpha }{m_{s}\ln\left(p_{cr}/p\right)f\left(\sigma_{m}/\sigma_{eq}\right)}\left[\dot{f}\left(\frac{\sigma_{m}}{\sigma_{eq}}\right)ln\left(\frac{p_{cr}}{p}\right)-f\left(\frac{\sigma_{m}}{\sigma_{eq}}\right)\frac{\dot{p}}{p}\right]$$

For the uniaxial loading case, Equation (6) can be written as:

(7) $$\dot{S}=\frac{N_{0}k_{0}\alpha }{m_{s}\ln\left(p_{cr}/p\right)}\cdot \frac{\dot{p}}{p}$$

Equation (7) describes the entropy increase rate for general mechanical process and the proposed model was successfully applied to the low-cycle fatigue life prediction of metallic materials [21]:

(8) $$\frac{N_{in}}{N_{f}}=-\frac{N_{0}k_{0}\alpha }{s_{cr}m_{s}}\left[\ln\left(\ln\left(\frac{p_{cr}}{p}\right)\right)-\ln\left(\ln\left(\frac{p_{cr}}{p_{th}}\right)\right)\right]$$

All the parameters in Equation (8) have clear physical meaning, where $N_{0}$ and $k_{0}$ are physical constants; α, $m_{s}$, $s_{cr}$, $p_{th}$, and $p_{cr}$ are parameters related to the material properties and can be obtained from experiments. It should be note that Equation (8) can also be applied in the accelerated fatigue test; the fatigue life can be obtained by the same initial cycles of fatigue with a well-determined database.

## 3. The Relation of Entropy Increase Rate and Normalized Creep Time

As the creep process is also an irreversible degradation process, Equation (7) can be applied in the creep process.

**Figure 1:**
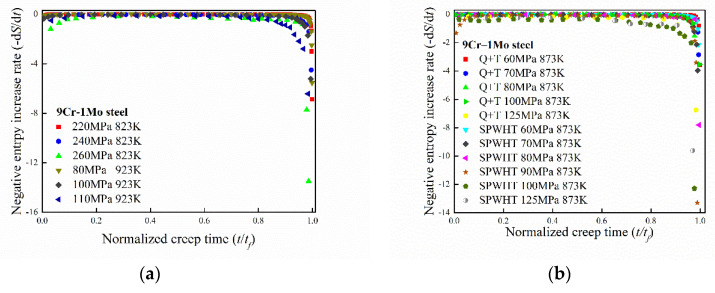

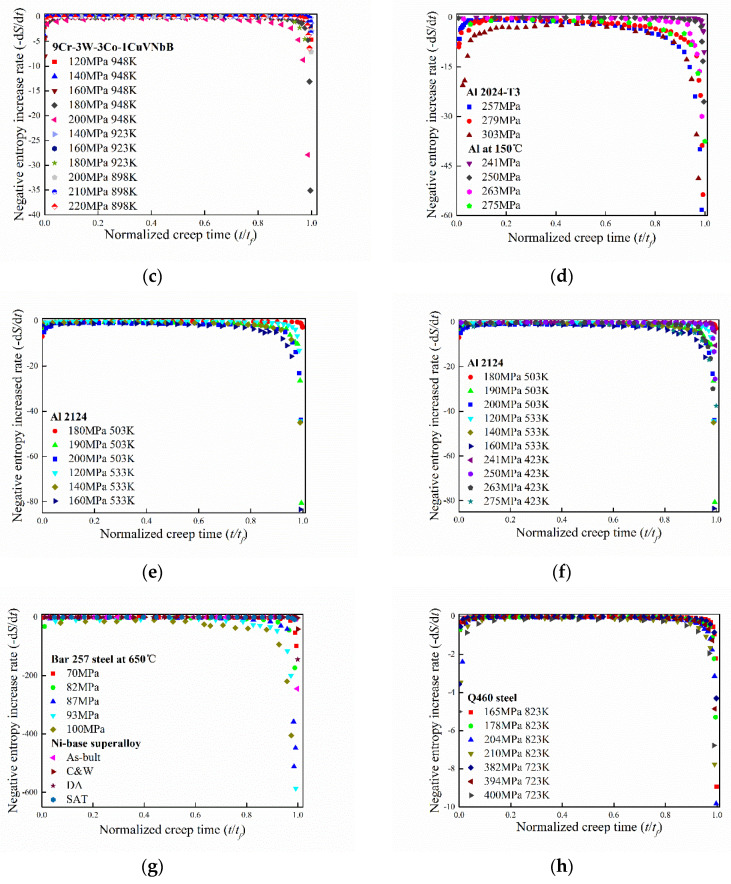
Change of the entropy increase rate with normalized creep time in the whole creep process: (**a**) 9Cr–1Mo steel at different temperatures; (**b**) 9Cr–1Mo steel under different treatment conditions (Q + T and SPWHT); (**c**) 9Cr–3W–3Co–1CuVNbB steel at different temperatures; (**d**–**f**) aluminum alloy at different temperatures; (**g**) Bar 257 steel and Ni-base super alloy at different temperatures; (**h**) Q460 steel at different temperatures.

To investigate the increase rate of entropy in the creep process, a wide variety of creep experimental data for metals and alloys from literature were adopted [29,30,31,32,33,34,35,36,37,38]. Detailed experimental data sources are listed in Table 1 and summarized as follows: Creep tests for 9Cr–1Mo steel were performed at different temperatures (500 °C, 550 °C, 600 °C, and 650 °C) under various stress levels from 80 MPa to 320 Mpa by using a uniaxial-load creep test frame [29,30]. Creep tests for 9Cr–3W–3Co–1CuVNbB were performed at different temperatures (625 °C, 650 °C, and 675 °C) and stress levels (120–220 MPa) by using creep machines (RDJ 50 CRIMS) [31]. Creep tests for aluminum alloys [32,33] were adopted to verify the proposed model. Creep tests for Bar 257 were performed at 650 °C with a stress range from 70 MPa to 100 MPa [34]. Creep tests of Ni-base superalloy were performed at different directions and heat treatments [35]. Creep tests of Q460 steel were carried out at nine temperatures in the range of 300–900 °C and at various stress levels ranging from 13 MPa to 509 MPa [36]. Constant load creep tests of Co–Cr–Mo alloy were conducted at a temperature range of 650–800 °C and a stress range of 240–330 MPa [37]. The creep samples of DZ125 were machined such that the applied stress is along the [001] orientation [38]. The creep entropy increase rate was determined by Equation (7). The cumulative plastic strain rate was obtained by taking the slope of figures, which contains two coordinate axes: Creep strain and creep time. Other parameters were determined in the previous research [21]. The negative entropy increase rate (−dS/dt) with normalized creep time (t/tf) for materials at different temperatures and applied creep stresses are shown in Figure 1.

As shown in Figure 1, the variation trend of entropy increase rate in creep process remains almost identical for different materials. In the early stage of creep, the dislocation multiplication and continuous movement lead to hardening of material. The entropy increasing rate decreases rapidly with time and then reaches a balance state. In the second stage, the creep strain rate achieves the minimum value and the entropy of the system increases at a fixed rate. In the last stage, the creep strain rate increases rapidly until the final fracture. The massive point defect separates out quickly at the grain boundary. The vacancy defect accelerates the creep strain rate and the final fracture. The entropy increasing rate of the system also increases rapidly in the last creep stage, which corresponds to increasing of the disorder degree of microstructure; the entropy increase rate attains infinity large when the final fracture occurs.

It should be noted that the entropy increase rate always keeps positive although its value reduces first and then increases; this phenomenon is consistent with the second law of thermodynamics. Comparison with experimental data shows that degeneration process during creep can be well represented by Equation (7).

To describe the variation trend of entropy increase rate with normalized creep time, an empirical formula is proposed based on the boundary features and the characteristics of Figure 1:

(9) $$\dot{S}=A\cdot \frac{1}{t}\cdot \frac{1}{\ln\left(t/t_{f}\right)}$$

where *A* is a parameter related to the constant creep stress, environment temperature, and material properties; $t_{f}$ is the time when final creep fracture occurs. The curve shape of Equation (9) shows sufficient similarity to ensure the characterization of entropy increase rate by continuous functions. 

**Figure 2:**
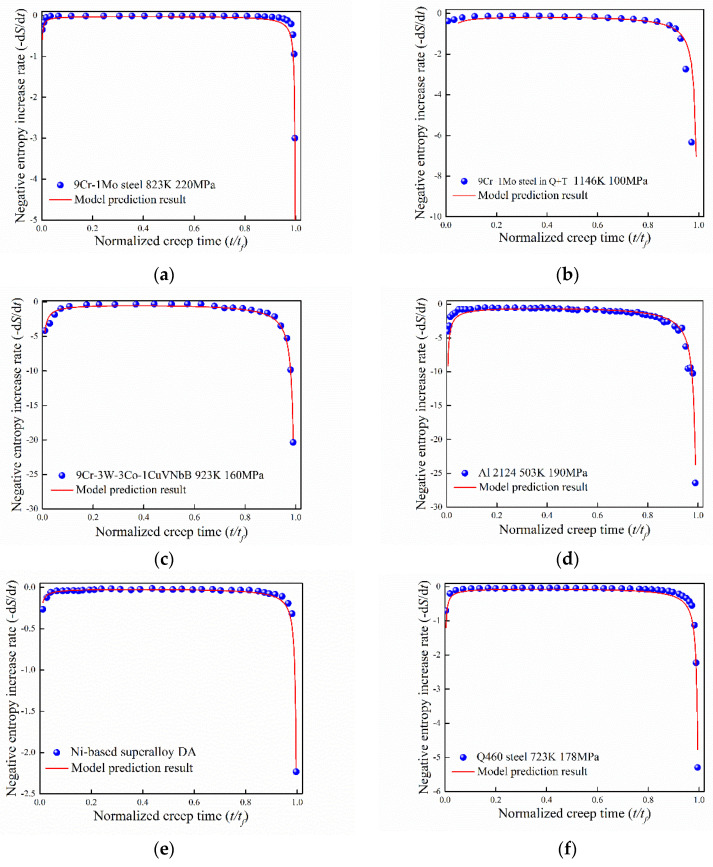
Verification of the proposed model compared with experimental data: (**a**) 9Cr–1Mo steel at 823 K with creep stress = 220 MPa [26]; (**b**) 9Cr–1Mo steel in quenched and tempered (Q + T) at 1146 K with creep stress = 100 MPa [27]; (**c**) 9Cr–3W–3Co–1CuVNbB at 923 K with creep stress = 160 MPa [28]; (**d**) Al 2124 at 503 K with creep stress = 190 MPa [30]; (**e**) Ni-based super alloy direct aging treatment at 718 °C/8 h/FC + 621 °C/10 h/AC [32]; (**f**) Q460 at 723 K with creep stress = 178 MPa [33].

Experimental data for different metallic materials have been adopted to verify Equation (9). For simplification, six group of experimental data for different material are shown in Figure 2. In general, the theoretical predictions agree well with the experimental data.

As shown in Figure 2, the entropy increase rate can be well described by Equation (9). The slight fluctuation of some experimental data points in the stable creep stage may originate from the deviation of data recording form reference. Entropy approaches infinity when the final fracture occurs, thus the change of entropy increase rate in the first creep stage is smaller than that of the third stage. While in the first stage, the entropy is limited although the entropy increase rate is discontinuous at the moment of applying stress. In the stable creep stage, the entropy increase rate approaches zero and this phenomenon is consistent for different experimental conditions. Thus, the increasing of entropy during creep process is related to the change of dislocation, which corresponds to the microstructure changes in the thermodynamic level.

## 4. The Entropy-Based Creep Strain Prediction Model

The change regulation of entropy increase rate during creep process is investigated. From Equations (7) and (9), an entropy-based creep strain rate prediction equation can be obtained:

(10) $$\dot{p}=\frac{Am_{s}p}{N_{0}k_{0}\alpha }\cdot \frac{1}{tln\left(t/t_{f}\right)}$$

Equation (10) can be regarded as an ordinary differential equation. After adjustment of Equation (10) and integral on both sides, the creep strain can be obtained:

(11) $$p=p_{cr}/\exp\left(\exp\left(\ln\left(\ln\left(\frac{p_{cr}}{p_{th}}\right)\right)-B\left[\ln\left(\ln\left(\frac{t_{f}}{t}\right)\right)-\ln\left(\ln\left(\frac{t_{f}}{t_{th}}\right)\right)\right]\right)\right)$$

(12) $$B=\frac{Am_{s}}{N_{0}k_{0}\alpha }$$

where $p_{th}$ is the initial value of cumulative plasticity in the microstructure of material, which represents the value at the beginning of creep damage accumulation; $p_{cr}$ is the threshold value of cumulative plastic variable when creep failure occurs. The corresponding creep time when $p=p_{th}$ and $p=p_{cr}$ are $t_{th}$ and $t_{f}$, respectively. The parameter B is related to the applied stress, temperature, and material properties. Equation (11) can be used to predict the creep strain during the creep damage process. 

To verify the developed model, experimental data for different metals and alloys were adopt for comparison. The main parameter in Equation (11) is B, which is related to the applied stress, temperature, and material properties such as elastic modulus. It can be obtained by fitting the experimental data through Equation (11). The threshold value of cumulative plastic strain and threshold time can be obtained from experiments. The initial value of cumulative plastic strain and creep time is determined by taking the first group of experimental data point and make the iterative operations. The predictions of the developed model are compared with experimental data for different materials in the following sections.

### 4.1. Ni-Base Super Alloy

**Figure 3:**
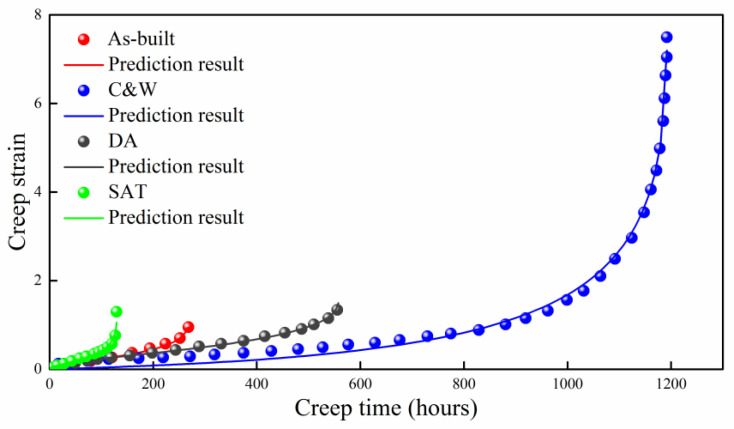
Prediction of Ni-base super alloy at different build directions and heat treatments. C&W: Cast-and-wrought; STA: Solution treatment and aging treatment; DA: Direct-aging treatment.

The creep properties of Ni-base super alloy at different build directions and heat treatments were studied experimentally by Kuo et al. [35]. Comparison of theoretical prediction with experimental data is shown in Figure 3. In general, the predictions agree well with experimental results. The applied stress (550 MPa) and temperature (923 K) remain unchanged for different test conditions. The main difference comes from the microstructure, which is reflected by different values of B in the developed model. However, it is difficult to effectively quantify microstructure for Ni-base super alloy at different build directions and heat treatments and establish the relation between B and microstructure.

### 4.2. Q460 Steel

**Figure 4:**
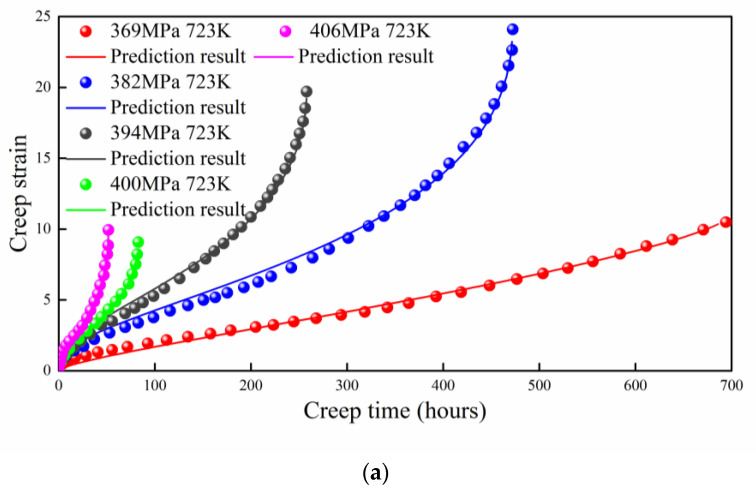

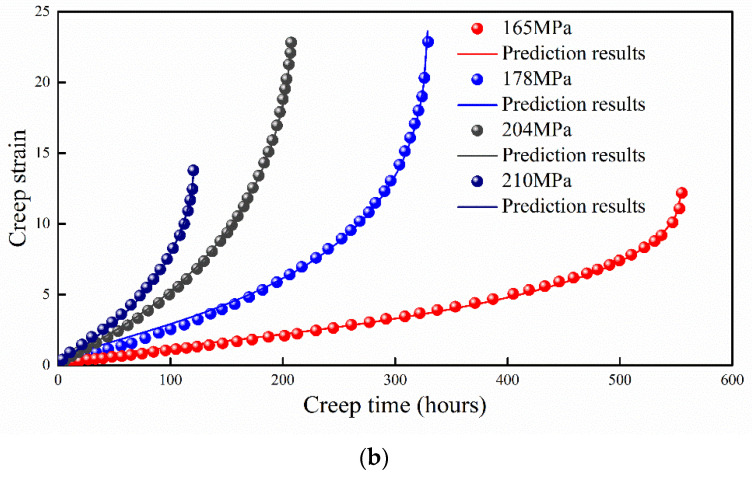
Prediction result of high strength Q460 steel at different temperatures; (**a**) 723 K; (**b**) 823 K.

The creep property of high-strength Q460 steel at different temperatures was studied by Wang et al. [36]. Decreasing of the maximum creep strain with the stress level was considered because sufficient plasticity can be developed with longer duration [36]. The maximum creep strain shows no obvious relation with creep stress for most of the cases. As shown in Figure 4, the prediction results agree well with the experimental data for both 723 K and 823 K cases. The absolute value of B is obtained by fitting the experimental data, which increases with the applied stress. The detailed discussion of B is given in the next section.

### 4.3. Bar 257 Steel at 923 K

**Figure 5:**
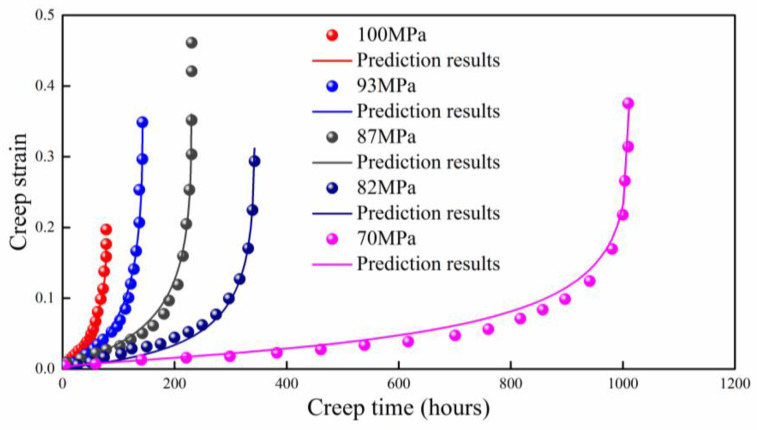
Prediction result of Bar 257 steel at 923 K.

As shown in Figure 5, the predictions of proposed model agree well with the experimental data for Bar 257 steel at 923 K [34], except for the applied stress of 70 MPa. The predicted creep strain is slight larger around the final fracture region. The absolute value of B also increases with the applied stress. The prediction with an applied stress of 87 MPa does not cover the last two data points as the prediction is composed of 1000 data points; this problem can be solved by making the data points denser (for instance, 3000 data points).

### 4.4. Al 2124 at 503 K and 533 K

**Figure 6:**
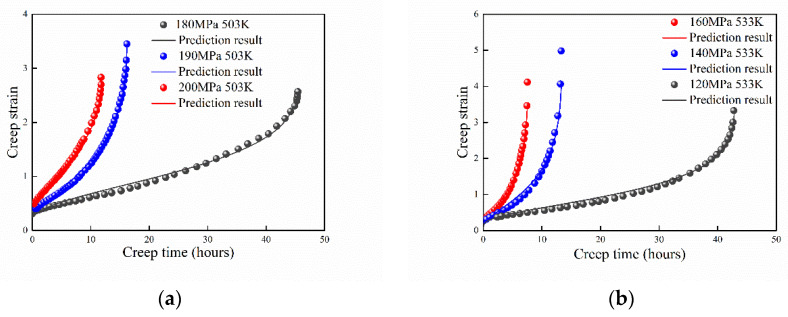
Prediction results of Al 2124 at (**a**) 503 K and (**b**) 533 K.

The high-temperature creep behaviors of Al 2124 at 473 K, 503 K, and 533 K were studied by Li et al. [33]. The theoretical prediction results are shown in Figure 6. Because the discontinuity of experimental data at 473 K (235 MPa) during the creep damage process, only the experimental data of 503 K and 533 K were selected to benchmark the proposed model. It shows that the prediction results agree well with the experimental data. With increasing of the applied stress, the absolute value of B increases as well.

### 4.5. Cr–1Mo Steel at 823 K and 923 K

**Figure 7:**
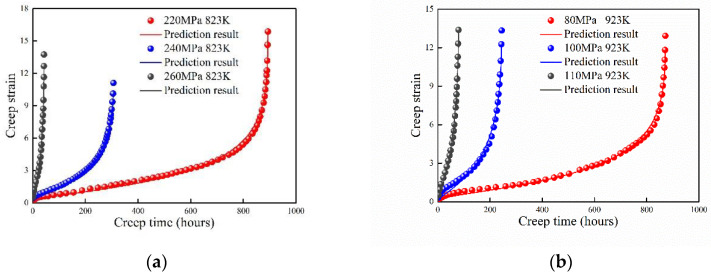
Prediction results of 9Cr–1Mo steel at (**a**) 823 K and (**b**) 923 K.

The creep behavior of modified 9Cr–1Mo steel at 823 K and 923 K were studied by Zhang et al. [30]. Comparison of the experimental data and prediction results are shown in Figure 7. The maximum creep strain approaches at 923 K for different stresses. However, this phenomenon was not observed for most of the other metals and alloys. Thus, it is hard to take the maximum plastic strain to evaluate the creep life [30]. The creep strain was well predicted with creep time by the proposed model. The absolute value of B increases with the applied stress.

### 4.6. Cr–3W–3Co–1CuVNbB Martensite Ferritic at 898 K, 923 K, and 948 K

**Figure 8:**
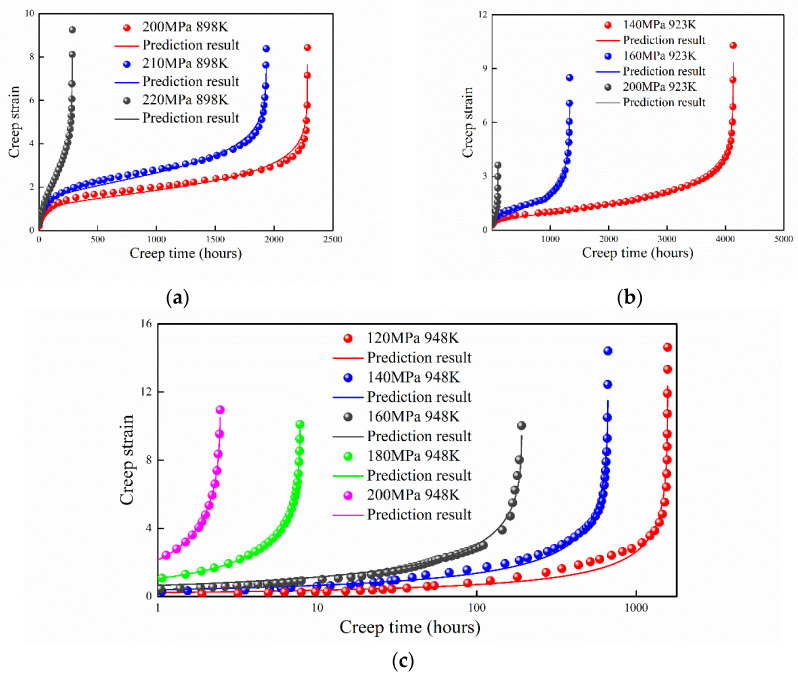
Prediction result of 9Cr–3W–3Co–1CuVNbB martensite ferritic steel at (**a**) 898 K, (**b**) 923 K, and (**c**) 948 K.

Xiao et al. [31] systematically investigated the creep behavior of 9Cr–3W–3Co–1CuVNbB martensite ferritic steel for a temperature range of 898 K to 948 K under uniaxial tensile stress from 120 to 220 MPa. As shown in Figure 8, predictions of the proposed model agree well with the experimental data. For the creep behavior of 9Cr–3W–3Co–1CuVNbB steel under 898 K, the first creep stage has a relatively longer time compared with other temperatures. It is difficult for the traditional models to predict the creep strain under these circumstances (it only maintains a certain precision in the first or third stages) [32]. The predictions of proposed model agree well with the experimental data except under the applied stress of 120 MPa at 948 K. Considering that the proposed model is single-parameter, the accuracy of prediction is acceptable.

### 4.7. Cr–1Mo Ferritic Steel in Quenched and Tempered (Q+T) and Simulated Post Weld Heat Treatment (Spwht) Conditions

**Figure 9:**
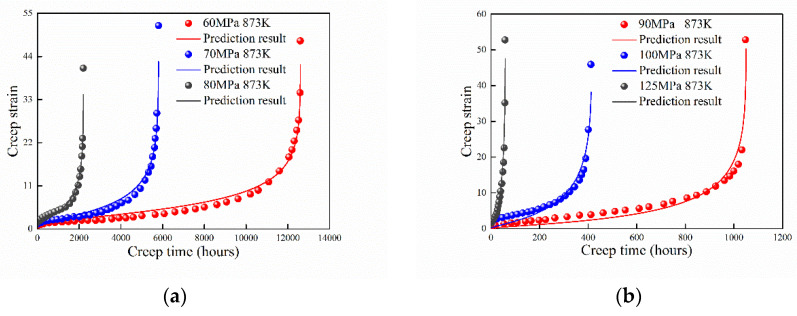

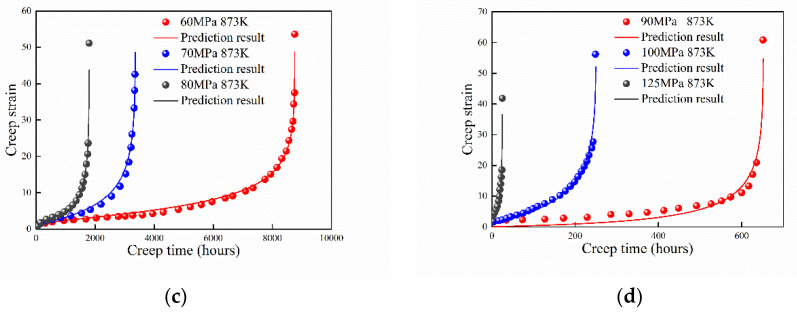
Prediction result of 9Cr−1Mo ferritic steel in quenched and tempered (Q + T) and simulated post weld heat treatment (SPWHT) conditions: (**a**) Q + T 60~80 MPa; (**b**) Q + T 90~125 MPa; (**c**) SPWHT 60~80 MPa; and (**d**) SPWHT 90~125 MPa.

The creep behavior of 9Cr–1Mo ferritic steel under SPWHT and Q + T treatment was studied by Choudhary [30]. The experimental data are compared with theoretical prediction, as shown in Figure 9. The predictions of proposed model agree well with the experimental data except under Q + T and SPWHT condition with the applied stress of 90 MPa. The second case is because the estimated $p_{th}$ is not converged. The prediction is obtained by setting $p_{th}$ as the first experimental data point. At the same time, the creep experiments usually accompanied with a certain degree of discreteness.

### 4.8. Co–Cr–Mo Alloy

**Figure 10:**
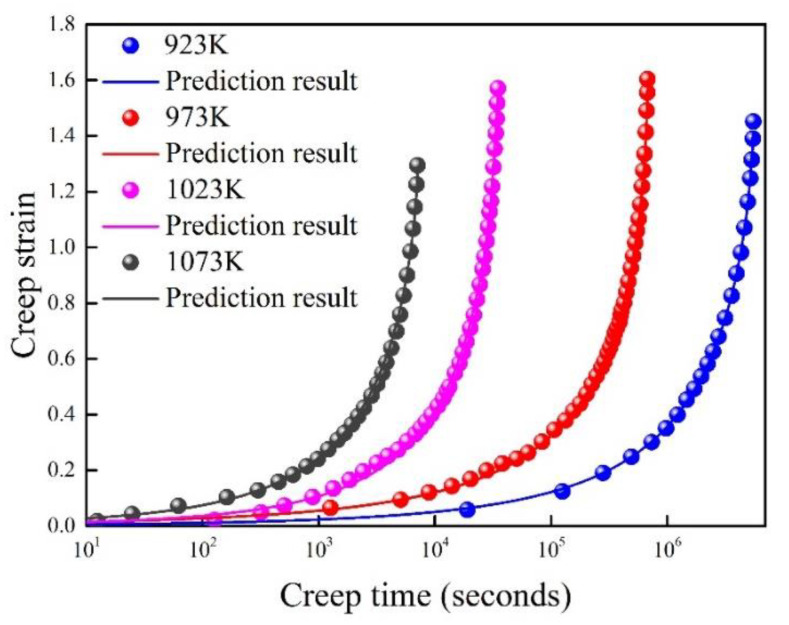
Prediction result of Co–Cr–Mo alloy at different ambient temperature.

The creep behavior of Co−Cr−Mo alloy at different temperatures (923 K, 973 K, 1023 K, 1073 K) was investigated by Sun et al. [37]. The experiments were carried out at a constant applied stress (240 MPa). The theoretical prediction is compared with experimental data, as shown in Figure 10. The transverse axes is taken as logarithmic coordinates to make the difference of experimental data more obvious. Generally, the prediction results agree well with the experimental data.

### 4.9. DZ125 Super Alloy

**Figure 11:**
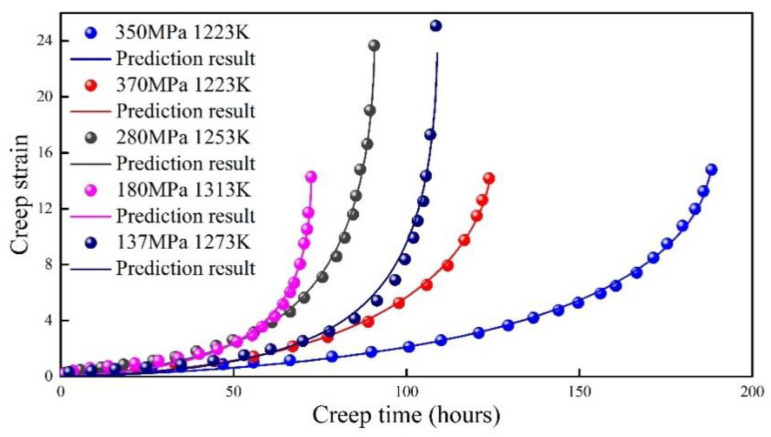
Prediction result of DZ125 super alloy at different creep stresses and temperatures.

The creep behavior of DZ125 super alloy at different creep stresses and temperatures was investigated by Fu et al. [38]. The theoretical prediction is compared with experimental data, as shown in Figure 11. The theoretical predictions agree well with the all five groups of experimental data. 

## 5. Parametric Analysis of the Proposed Model

**Figure 12:**
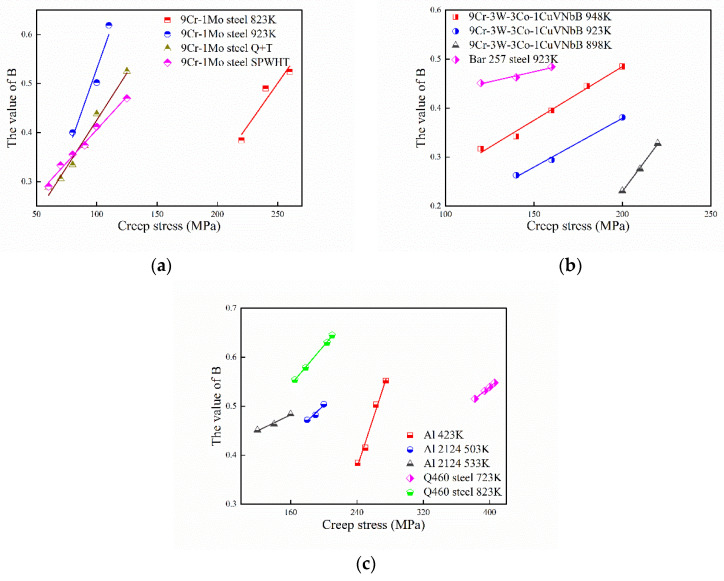
The relation between the parameter *B* and applied stress. (**a**) 9Cr-1Mo steel; (**b**) 9Cr-3W-3Co-1CuVNbB and Bar 257 steel; (**c**) Al and Q460 steel.

As is well known, the creep strain is strongly influenced by the applied stress and temperature. The creep strain will increase with higher applied stress and temperature. These effects cannot be ignored in the creep analysis. Therefore, the parameter B in Equation (11) should be associated with the applied stress and temperature. Assuming that these two effects are irrelevant, which is commonly accepted in the traditional models [39], the parameter B should have a form as follows:

(13) B=M·f(σ)·g(T)

where *M* is a coefficient that contains α, $m_{s}$, $k_{0}$, and $N_{0}$. The relation between *B* and applied stress are verified by the experimental data in Section 4. The values of *B* and its linear fitting with applied stress are shown in Figure 12:

As shown in Figure 12, with increasing of the applied stress, the value of B also shows a near-linear increasing trend for different materials. In the current study, the value of B has a relative concentration range of 0.2~0.7 (maximum = 0.645 for Q460 steel at 210 MPa 823 K; minimum = 0.231 for 9Cr–3W–3Co–1CuVNbB at 210 MPa 898 K).

**Figure 13:**
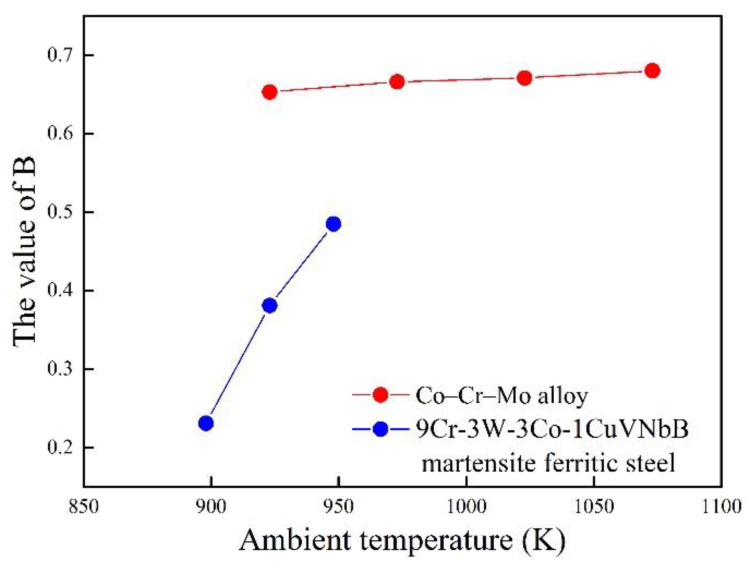
The relation between the parameter *B* and temperature.

The relationship between *B* and temperature is shown in Figure 13. With the increase in temperature, the value of B also increases for 9Cr–3W–3Co–1CuVNbB and Co–Cr–Mo alloy. Although the relationship between *B* and temperature approaches linear for Co–Cr–Mo alloy, determination of an accurate relationship still requires more experimental data.

## 6. Conclusions

Based on the continuum damage mechanics and the statistical definition of entropy, the entropy increasing rate during creep process is investigated. The conclusions are summarized as follows:

1. The entropy increasing rate for creep is investigated with experimental data for different metallic materials. Similar entropy increasing trend is observed with normalized creep time. A theoretical model is proposed to describe the relationship based on the characteristic of boundary conditions. Comparison with experimental data shows that the developed model gives reasonably accurate estimation of entropy increasing rate in the creep process.
2. An entropy-based creep strain prediction model is proposed with respect to the entropy increasing rate. Predictions of the proposed model agree well with the experimental data for different metallic materials.
3. The single parameter B in the proposed model is associated with the applied stress and temperature. In general, the parameter increases linearly with the applied stress.

## Figures and Tables

**Table 1:** The experimental data adopted in the current study.

Materials	Experimental Data Sources
9Cr–1Mo steel	[29,30]
9Cr–3W–3Co–1CuVNbB martensite ferritic steel	[31]
Aluminium alloy at 150 °C	[32]
Al 2024-T3	[33]
Al 2124	[33]
Bar 257 steel at 650 °C	[34]
Ni-base superalloy	[35]
Q460 steel	[36]
Co–Cr–Mo alloy	[37]
DZ125 super alloy	[38]
